# In vitro exposure of simulated meat-cooking fumes to assess adverse biological effects

**DOI:** 10.1038/s41598-017-11502-8

**Published:** 2017-09-07

**Authors:** Bijay Kumar Poudel, Jungwook Choi, Jae Hong Park, Kyung-Oh Doh, Jeong Hoon Byeon

**Affiliations:** 1 School of Mechanical Engineering, Yeungnam University, Gyeongsan, 38541 Republic of Korea; 2 School of Health Sciences, Purdue University, West Lafayette, IN 47907 United States; 3 Department of Physiology, Yeungnam University, Daegu, 42415 Republic of Korea

## Abstract

The heterocyclic amine 2-amino-1-methyl-6-phenylimidazo[4,5-b]pyridine (PhIP) is considered as a human carcinogenic or mutagenic compound that is produced from the co-condensation of creatinine and amino acids as meats cook at high temperatures. The cooking of meats at high temperatures produces fumes, and these fumes can be suspended as aerosols via the vapor-to-particle (or -droplet) process in a temperature gradient field. Size distributions of the aerosols included a significant portion of nano- and submicron-sized particles, and these can be directly deposited in the lungs and on skin by particle transport phenomena near cooking areas. In this study, for the first time, PhIP-incorporated oleic acid (OA, simulating cooking oil) (PhIP@OA) particles, including individual particulate PhIP as simulated fumes from meat cooking, were constantly produced via collison atomization and subsequent drying processes. The aerosol particles were then dispersed in phosphate-buffered saline for cytotoxicity and senescence-associated *β*-galactosidase assays, which were compared with dissolved PhIP in dimethyl sulfoxide. PhIP and PhIP@OA did not show significant cytotoxic effects on SHSY5Y, MRC5, and human dermal fibroblast cells compared with the dissolved PhIP but clearly induced premature senescence activities that may be caused by a limited release of PhIP molecules from the particulate PhIP.

## Introduction

By the year 2050, premature deaths from gaseous and particulate air pollutants will be significantly higher than those from human immunodeficiency virus and malaria^1^. The adverse effects of fine particulate matters on human health in indoor environments also are receiving much attention because people are spending more time indoors (up to 90%)^2^. In particular, cooking fumes have been introduced as a major indoor source of fine-particle organic aerosols containing hazardous chemical substances (e.g., hydrocarbons, fatty acids, and mist)^3, 4^; thus, human exposure to these substances could increase carcinogenic risks^5^. When meats cook at high temperatures, a deposit of these organic aerosol forms on the surfaces of kitchen walls and ventilation fans, which then leads to potential adverse effects on indoor air quality and inhabitant health^4, 6^. People are at a significant risk of exposure (i.e., pulmonary and dermal) to these aerosols because the aerosol emissions from meat cooking have been measured at 40 g per kg meat^7^.

Emissions from the thermal cooking of meats contain polycyclic aromatic hydrocarbons (PAHs) and heterocyclic aromatic amines (HCAs) because both compounds are produced during high temperature treatments of proteinaceous materials^8, 9^. The heterocyclic amine 2-amino-1-methyl-6-phenylimidazo [4,5-b]pyridine (PhIP) is one of the representative HCAs and a by-product of Maillard or browning reactions at high temperatures among free amino acids, creatine/creatinine, and sugars during meat cooking^9^. PhIP is known to react with DNA to produce carcinogenic adducts^10^; thus, it can cause an increase in the incidence of cancers from dietary exposure^11, 12^. Recently, the neurotoxic effects of PhIP were investigated *in vitro*, and results showed toxic effects to dopaminergic neurons due to oxidative stresses^13^. Expression of cytochromes regarding metabolic and toxicological aspects was also introduced for different cell lines (i.e., neuronal and skin) upon the cell exposure to PhIP^14, 15^. PhIP was detected in aerosols (i.e., smoke condensate) produced during the frying of beef patties and bacon^16^. Meat cooking at high temperatures generates oil mist aerosols from the cooking oil and/or animal fat, and PhIP aerosols may be incorporated in the oil mist as well as in carbonaceous particles (e.g., black soot)^17, 18^. However, there are no appropriate test protocols or relevant data for the biological effects of pulmonary and dermal exposures to these aerosol particles.

**Figure 1:**
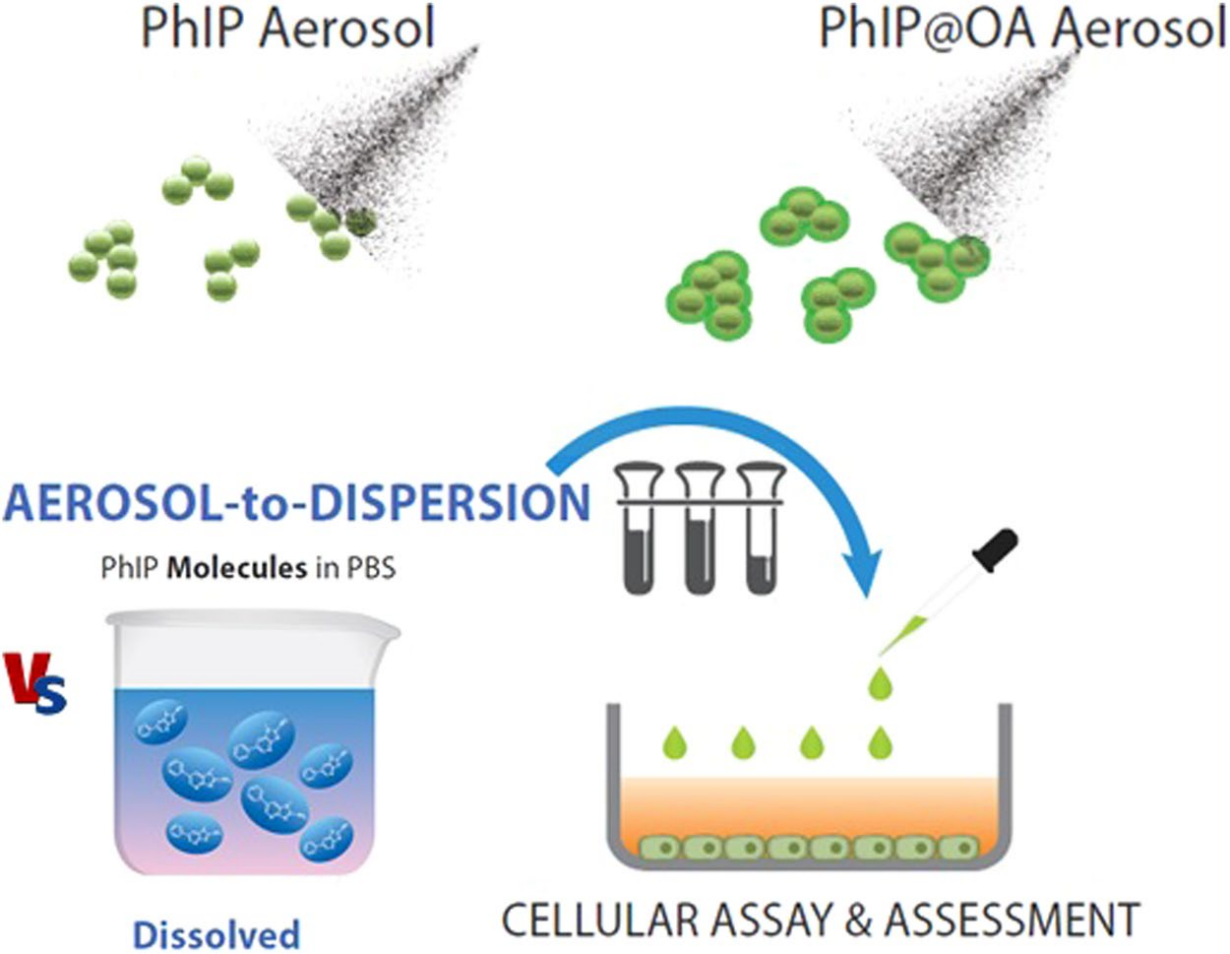
Schematic of fabrications of PhIP and PhIP@OA aerosols via the collison atomization of PhIP (or PhIP and OA) dissolved in DMSO solution. The fabricated aerosols were dispersed in PBS solution to be applied to *in vitro* assays (MTT, SA-*β*-gal, western blot, and flow cytometry) and compared with dissolved PhIP.

In the present study, we attempted to produce a constant supply of PhIP or oleic acid (OA)-incorporated PhIP (PhIP@OA) aerosol particles using a serial aerosol reactor comprising a collison atomizer and a diffusion dryer. PhIP (or PhIP@OA) was dissolved in dimethyl sulfoxide (DMSO) to form aerosol droplets, and the droplets were then passed through a diffusion dryer to extract DMSO, thereby forming particulate PhIP aerosols. OA was frequently employed as a representative aerosol component from meat cooking because organic aerosols emitted from meat cooking contain a significant fraction of OA^19, 20^. The collected PhIP aerosols were dispersed in phophate-buffered saline (PBS) at the chosen mass concentration for biological assessments. Cytotoxicity measurements of the dispersion were conducted using the colorimetric 3-(4,5-dimethylthiazol-2-yl)-2,5-diphenyltetrazolium bromide (MTT) assay for cell metabolic activities in SHSY5Y (human neuroblastoma), MRC5 (human lung fibroblast), and human dermal fibroblast (HDF) cells, which then were compared with dissolved PhIP (i.e., solute ion state), as shown in Fig. 1. Western blot analysis with senescence-associated beta-galactosidase (SA-*β*-gal) and reactive oxygen species (ROS) generation assays were further conducted on HDF cells to correlate with the cytotoxicity assays. Dioctyl sebacate (DOS) was also employed instead of OA to produce PhIP@DOS aerosol particles using the atomizer. Spark-produced carbon black (CB, simulating cooking soot) particle-laden flow was employed as an operating gas to atomize the PhIP@OA solution as an effort to fabricate another realistic configuration (PhIP-CB@OA) of PhIP-containing aerosol particles (Supplementary Fig. S1) for the evaluation of their adverse effects.

## Results

### Physicochemical Characterizations

**Figure 2:**
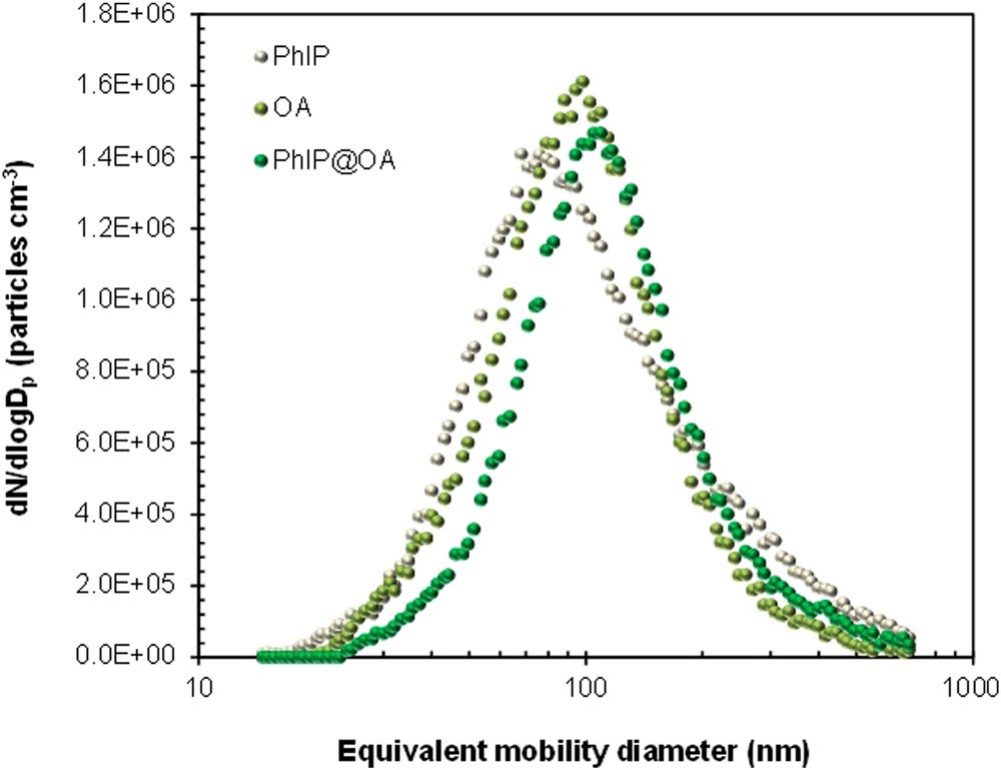
Particle size distributions of PhIP, OA, and PhIP@OA aerosols. Size distributions were measured using SMPS, which comprised an aerosol classifier (3082, TSI, USA), differential mobility analyzer (3081A, TSI, USA), condensation particle counter (3772, TSI, USA), and aerosol charge neutralizer (XRC-05, HCT, Korea). Data acquisition was performed using the AIM3938 software (TSI, USA). Summarized results are described in Supplementary Table S2.

Size distributions of aerosolized PhIP and PhIP@OA particles were measured using SMPS (Fig. 2), and the details of distribution are summarized in Supplementary Table S1. The size distribution of OA droplets was also measured for comparison with PhIP@OA particles. According to previous reports^21, 22^, aerosol particle sizes (*D*_p_) from meat cooking at high temperatures were within nano- and submicron-ranges even with cooking oils. The number concentrations (*N*_p_) of aerosols from meat cooking processes ranged from 10^5^ to 10^6^ particles cm^−3^ ^21–23^. To prepare simulated PhIP (or PhIP@OA) aerosols with size distributions similar to previous reports, the concentration of PhIP (or PhIP@OA) dissolved in DMSO solution was controlled based on the following equations:

1 $${D}_{p}=\sqrt[6]{\frac{{\rho }_{p}}{{\rho }^{\ast }}}\sqrt[3]{\frac{{C}_{s}}{{\rho }^{\ast }}}{D}_{d}$$

2 $${N}_{p}=\frac{6\dot{m}}{{\rho }_{p}{D}_{p}^{3}Q}$$

where *ρ*_p_ and *ρ* ^*^ are the densities of particles and reference (1 g cm^−3^), respectively; *C*_s_ is the concentration of PhIP (or PhIP@OA) dissolved in DMSO; *D*_d_ is the droplet diameter [measured using aerodynamic particle sizer (3320, TSI, USA)]; $\dot{m}$ is the mass production rate of particles [measured using a piezobalance dust monitor (3522, Kanomax, Japan)]; and *Q* is the gas flow rate. By modulating the solution concentration, appropriate size distributions (Supplementary Table S1) were secured for further characterizations and biological assays. The size distribution of PhIP shifted to larger sizes when OA was incorporated, and interestingly, there were no other peaks, although OA aerosols have a size distribution different compared with PhIP. This implies that OA was nearly quantitatively incorporated with PhIP to be a single particle during the atomization and subsequent drying processes. Analogous characteristics could be seen in incorporations of PhIP with other compounds such as PhIP-CB@OA (to simulate cooking soot containing fumes by further adding CB) and PhIP@DOS (to simulate another cooking oil), as shown in Supplementary Figs S2 and S3, and Table S3. This implies that atomization and subsequent drying processes may be suitable for preparing simulated meat cooking fumes, and a serial combination using an CB particle generator further showed feasibility for preparing soot-containing (from meats or cooking fuels) composite-like cooking fumes.

**Figure 3:**
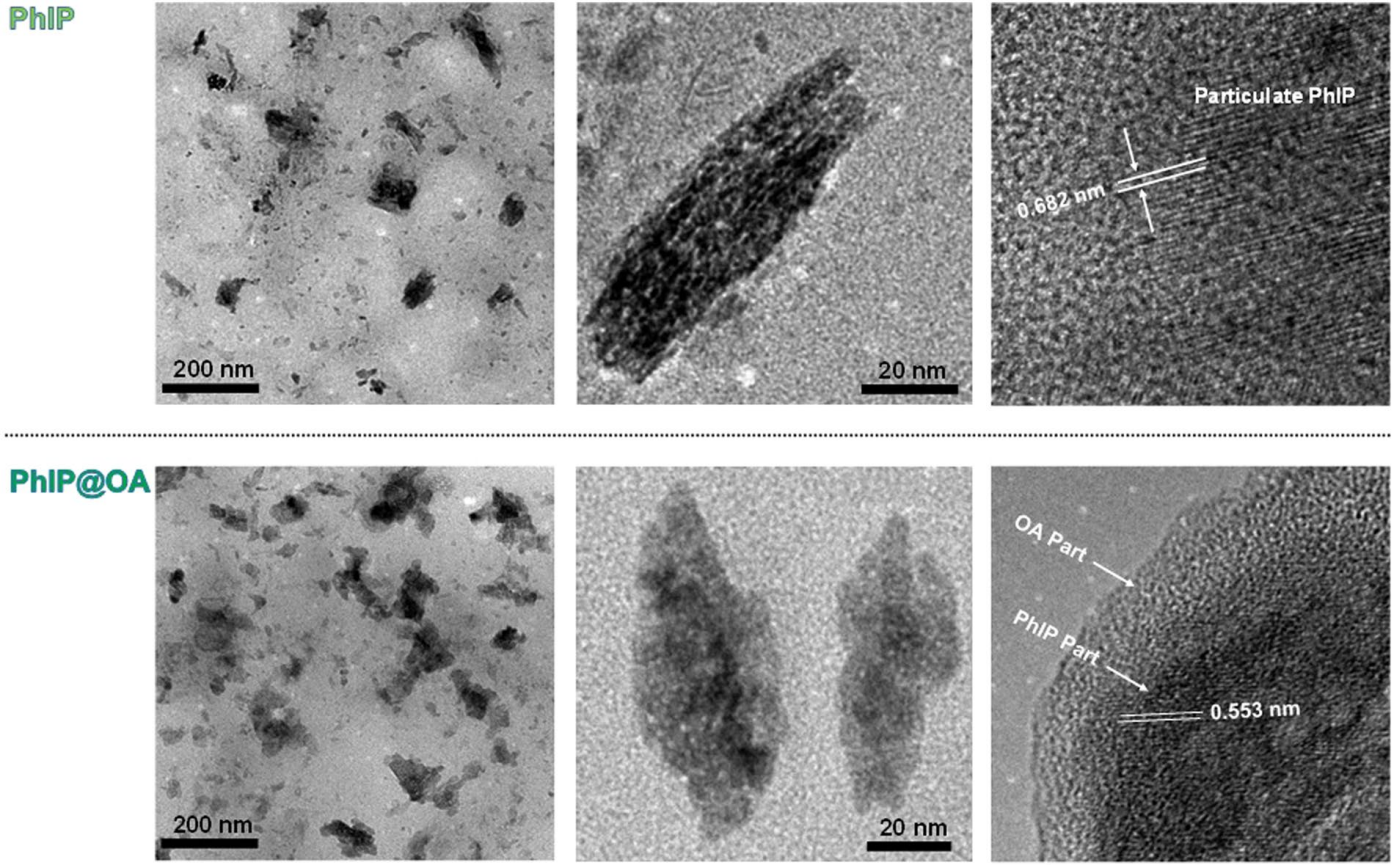
Low– and high–magnification TEM images of PhIP and PhIP@OA aerosols. Specimens were prepared by direct aerosol deposition of particles on carbon-coated copper grids (Tedpella, USA) using a Mini-Particle Sampler (Ecomesure, France).

Using TEM measurements (Fig. 3), we confirmed that atomization and subsequent drying processes of dissolved PhIP (or PhIP and OA) produced PhIP (or PhIP@OA) particles. The TEM micrographs of PhIP showed anisotropic irregular morphologies with a size of 71 ± 5.4 nm. Interestingly, the high-magnification TEM image displayed lattices with a gap distance of 0.682 nm, which may be consistent with polycarbonate crystallites from the solidification of carbonaceous precursors^24^ that showed larger lattice fringes (>0.54 nm) than graphitic carbon. Anisotropic morphologies were also found in case of PhIP@OA, and the anisotropic structures were covered by a lighter contrast layer that was caused by OA incorporation on particulate PhIP. Lattice fringes also existed in the core region (i.e., the primary PhIP section) of the particulate PhIP@OA. The lattice distance was 0.553 nm, and this was different from that measured in particulate PhIP alone, implying that the polycarbonate crystallization could be affected by co-existing compounds during the solidification process into particles. This tendency could also be seen in other configurations (i.e., PhIP-CB@OA and PhIP@DOS; Supplementary Fig. S4). In addition, in case of PhIP-CB@OA, graphitic structures (showing a lattice distance of 0.33 nm) co-existed at the core region of the particles. This is consistent with a previous report^25^ proving that pre-formed particles injected into a droplet containing other solute compounds can be encapsulated by the solutes during solvent extraction.

To validate the surface chemistry of particulate PhIP, FTIR measurements were performed using directly sampled aerosol PhIP particles on polytetrafluoroethylene substrates (Supplementray Fig. S5). The particulate PhIP showed characteristic bands at 790 cm^−1^, 1430 cm^−1^, and 1665 cm^−1^ corresponding to the pyridine ring. The bands at 1105 cm^−1^ and 1635 cm^−1^ could be assigned to the –N=N– and the aromatic C–H, respectively, and the presence of these bands implied that the aerosol PhIP particles had characteristic groups of PhIP. On the other hand, these characteristic bands disappeared when OA (or DOS) was incorporated; only the bands for OA (or DOS) remained for the PhIP@OA (or PhIP@DOS) sample and for the PhIP-CB@OA sample. In PhIP@OA and PhIP-CB@OA samples, for example, the bands at around 1470 cm^−1^ were attributed to the in-plane –O–H groups of OA, and an intense band at 1710 cm^−1^ was related to the carbonyl stretching mode. PhIP@DOS showed an intense band at 1720 cm^−1^ corresponding to the carbonyl stretching vibration. These findings suggest that OA (or DOS) was deposited uniformly over the entire surface of all particulate PhIPs.

### Biological Assays

**Figure 4:**
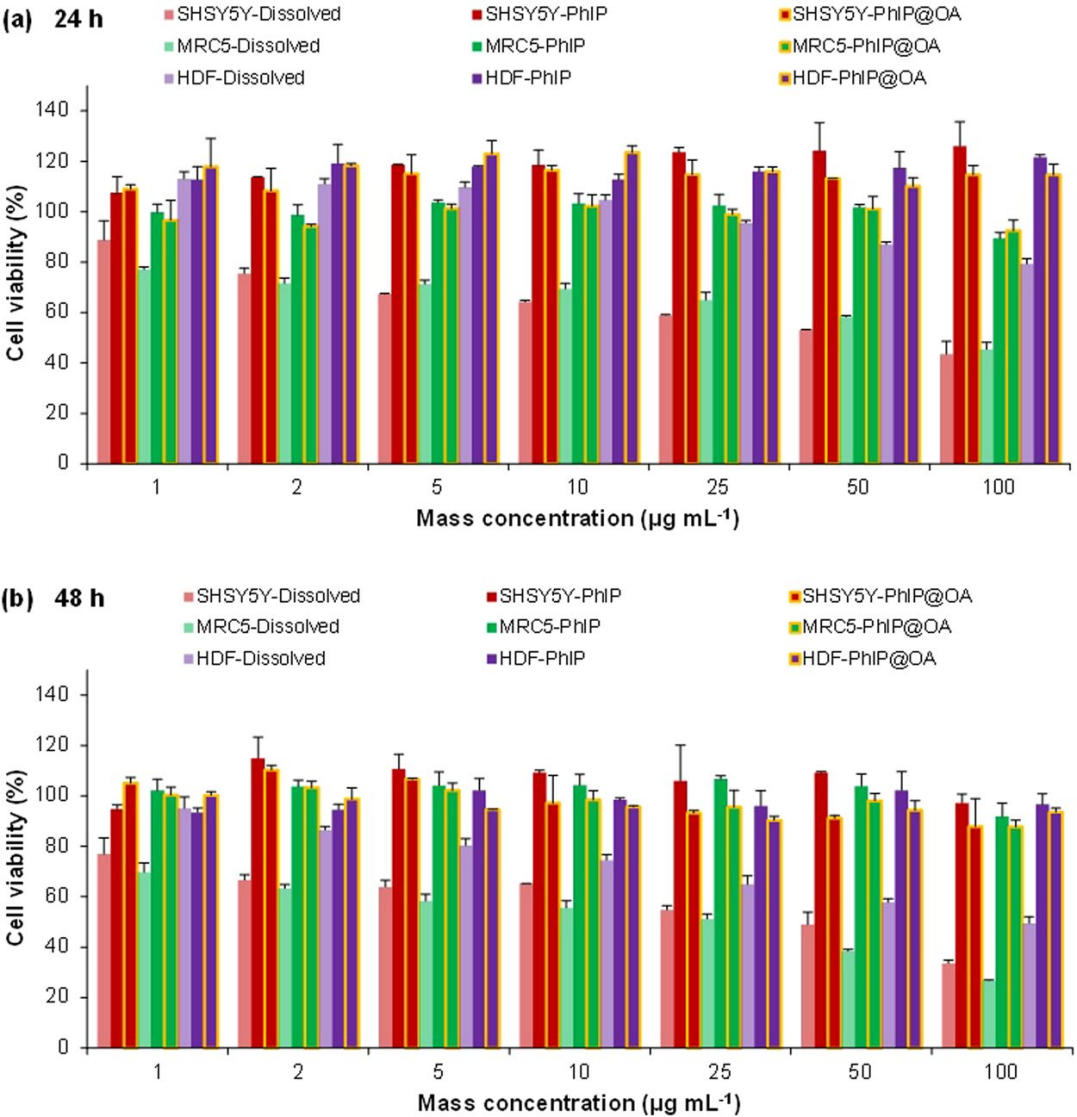
MTT assay results (*p* < 0.05) for PhIP and PhIP@OA aerosols on SHSY5Y, MRC5, and HDF cells after (**a**) 24 h and (**b**) 48 h exposures in a concentration-dependent manner (1–100 μg mL^−1^). Cell viabilities after aerosol exposures are compared with those after dissolved PhIP (i.e., not a dispersion) exposure.

The simulated aerosol particles were electrostatically collected on a cylindrical collection rod after passing through a diffusion dryer. Particles were then dispersed in PBS under ultrasonic irradiation to be employed for particle size measurements (Supplementray Fig. S6) and *in vitro* assays. The DLS measurement showed nano and submicron size distributions for these simulated particles, and the particles tended to increase in size when OA or DOS was incorporated, which was consistent with SMPS measurements. The slightly larger size distributions in DLS measurement might be due to coincidence effect of particles in dispersion during the light scattering because of significantly higher concentration of particles in PBS than those in gas (SMPS). Successive measurements of zeta potentials showed negative polarities for the dispersed simulated particles in PBS (pH 7.4), and there were no significant differences among the samples (Supplementary Table S4). Based on MTT assays, there were significant differences between the dissolved and particulate PhIP (PhIP alone and PhIP@OA) samples in all cell lines, although the range of mass concentration (1–100 µg mL^−1^) was identical. Dissolved PhIP showed greater cytotoxicities at all concentrations after 24 h (Fig. 4a) and 48 h (Fig. 4b) incubations, and cancerous cells (i.e., SHSY5Y and MRC5) were more sensitive compared with HDF cells. This might be caused by cell type-dependent toxic effects of PhIP. A previous report proved this phenomenon based on genotoxic DNA fragmentation by comparing cytotoxicities between carcinoma cells (HepG2 and Caco2 cells) and normal adipocytes^26^. On the other hand, remarkable cytotoxic effects could not be seen in particulate PhIP and PhIP@OA samples even after the 48-h incubation. Other particulate samples (i.e., PhIP-CB@OA and PhIP@DOS) produced similar results even after extended incubations (96 h) (Supplementary Fig. S7). This may be due to remarkably smaller releases of PhIP molecules from the particulate PhIP and was confirmed by high-performance liquid chromatography (HPLC) measurements. The intensity of PhIP molecules from the particulate PhIP was significantly weaker compared with that of those from the dissolved PhIP (more than 62 times at least) (Supplementary Fig. S8), and this may have been caused by the polycarbonate crystallization of PhIP during the aerosolization (refer to Fig. 3).

**Figure 5:**
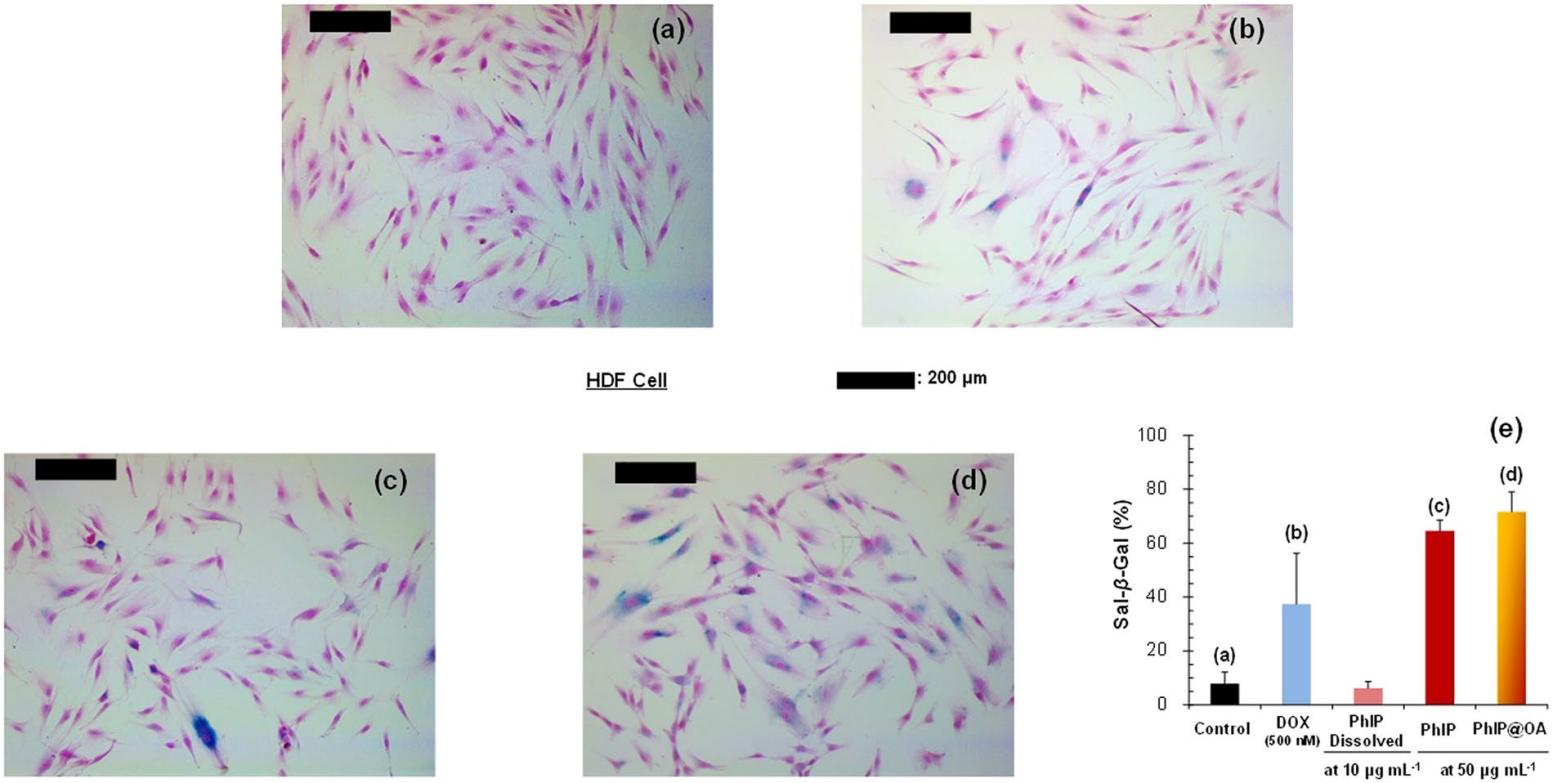
SA-*β*-gal assay for HDF cells. Optical microscope images of (**a**) control, (**b**) DOX (500 nM)-treated, and (**c**,**d**) PhIP and PhIP@OA aerosols (50 μg mL^−1^). (**e**) Percentages of SA-*β*-gal positive cells after treatments with PhIP and PhIP@OA aerosols, including DOX and dissolved PhIP for comparative purposes. Dissolved PhIP was applied at 10 μg mL^−1^ because significant cell death occurred when 50 μg mL^−1^ was applied.

**Figure 6:**
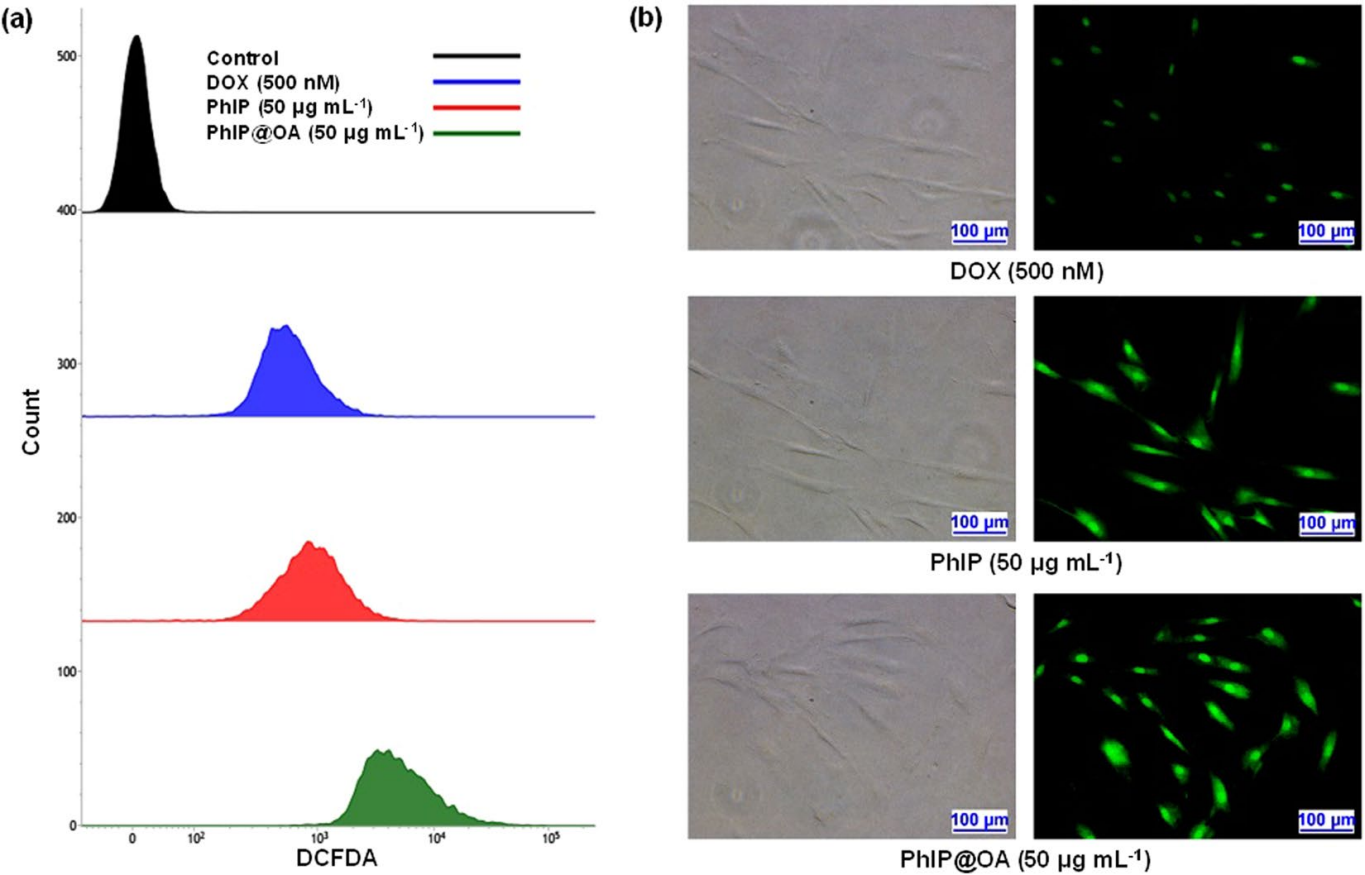
ROS generation measurements via DCFDA detection using flow cytometry and fluorescent microscopy. (**a**) DCFDA assays to measure intracellular ROS generations (DCF induced green fluorescence intensity) in HDF cells after treatments with PhIP and PhIP@OA particles (50 μg mL^−1^ for each) and DOX (500 nM). (**b**) Evaluations of ROS generation in HDF cells from DCFDA assay using fluorescence microscopy.

According to MTT assays, the particulate PhIP did not show remarkable cytotoxic effects on the chosen cell lines even for further OA, CB@OA, or DOS incorporation. Considering the significantly smaller releases of PhIP molecules from particulate PhIP, the SA-*β*-Gal assay was employed to assess the adverse effects of low-level PhIP exposures to HDF cells (Fig. 5). A previous study reported that low-level PhIP can introduce perturbations from inflammation and physiological conditions (diabetes, cancer, obesity, etc.), suggesting that the effects of low-level PhIP exposures are complex and different compared with those of high-level exposures^27, 28^. To evaluate the effects of low-level PhIP exposures, a low-concentration DOX (500 nM) was employed as a control in SA-*β*-Gal assays to compare its activity with that of the particulate PhIP samples because low-concentration DOX is known to induce premature senescence in HDF cells^29^. DOX and particulate PhIP samples (50 µg mL^−1^) induced SA-*β*-Gal positive cells, showing bluish stains on the cells (Fig. 5b–d); particulate samples showed higher SA-*β*-Gal activities (>1.8 times) than DOX. On the other hand, dissolved PhIP (10 µg mL^−1^) showed an activity (Fig. 5e) only comparable to the control (Fig. 5a), and this supports the premise that different activities occur with low-level PhIP than high-level PhIP exposures. To corroborate the observation of premature senescence induction by the particulate PhIP, western blotting and DCFDA assay were employed to analyze the upregulation of p21 and p53 pathways^30^ and ROS generation^31^, respectively. The western blot analysis showed elevated p21 and p53 expression from PhIP samples compared with DOX, which was consistent with the SA-*β*-Gal assay results (Supplementary Fig. S9). Based on DCFDA assays, ROS levels in HDF cells significantly increased after exposures to particulate PhIP and PhIP@OA samples (Fig. 6a), and the generation of HDF cells was further confirmed by fluorescence microscope measurements (Fig. 6b). Based on the combination of results from these assays, it can be inferred that slow releases of PhIP molecules from particulate PhIP samples may affect the induction of senescence in HDF cells, although particulate samples did not show significant cytotoxic effects in MTT assays. This implies that further studies will be required to assess the acute and chronic exposures of aerosol PhIP and the related particles at an *in vivo* level.

## Discussion

PhIP is one of the HCAs found in meat cooked at high temperatures and has carcinogenicity and mutagenicity; thus many studies have been conducted to evaluate its adverse effects at *in vitro* and *in vivo* levels. However, exposures and the biological effects of aerosolized PhIP have never been considered, although meat cooking produces large amount of nano- and submicron-sized aerosol particles. Based on the physicochemical characterizations, controlling the atomization conditions can produce simulated PhIP and its related particles (PhIP@OA, PhIP-CB@OA, and PhIP@DOS) with nano and submicron size distributions (~10^6^ particles cm^−3^); this is well-matched to the distributions of fumes from the actual meat cooking processes. The TEM measurements of particulate PhIP presented polycarbonate crystallites, and the lattice fringes of the crystallites also were found for the cases where OA and DOS were incorporated into the molecules. Nevertheless, particulate PhIP maintained the characteristic IR bands of standard PhIP, and these bands disappeared when OA or DOS were included in the PhIP solution (i.e., PhIP dissolved DMSO) for atomization. This implies that the surfaces of particulate PhIP were uniformly covered by OA or DOS component during the atomization and subsequent drying processes; this was confirmed by TEM measurements. The same was true for PhIP-CB@OA samples in which CB was introduced to the molecules.

There were significant differences between the dissolved and particulate PhIP samples in cytotoxicity profiles that may be caused by slow releases of PhIP molecules from the particulate PhIP that has a polycarbonate crystalline structure. The quasi-crystalline nature induced a significantly smaller elution of PhIP molecules into PBS, which was proven by HPLC measurements. Adverse effects from the low-level PhIP releases were evaluated through SA-*β*-Gal assays, and these were compared with those of 500 nM DOX releases. Unlike MTT assays, particulate PhIP and PhIP@OA induced senescence activities in HDF cells that were comparable to those induced by DOX as a control. The dissolved PhIP (10 µg mL^−1^) did not show remarkable senescence activities, and this supports the premise that different cellular responses could result from different PhIP exposure levels (i.e., low and high concentrations of PhIP molecules). Western blot analysis, the DCFDA assay, and fluorescence microscopy further supported the results from SA-*β*-Gal activities for low-concentration DOX and particulate PhIP. From these *in vitro* assessments for simulated meat cooking particles, further studies will be required to evaluate the adverse effects regarding acute and chronic exposures at *in vivo* levels. This is necessary to secure more useful information from more efficient analyses of PhIP aerosol exposures and its related configurations.

Collison atomization-based processing was designed and employed to produce simulated PhIP and its related aerosol particles with size distributions similar to fumes from high temperature meat cooking. The particulate PhIP from the aerosol process showed a quasi-crystalline structure of polycarbonate, and this structure induced significantly smaller releases of PhIP molecules from particulate PhIP surfaces. The crystalline nature of particulate PhIP was retained when other components (OA, CB@OA, and DOS) were introduced during the aerosolization. This property resulted in remarkable differences between the dissolved and particulate forms of PhIP in cytotoxic profiles for SHSY5Y, MRC5, and HDF cells. As a result, biological assays for low-concentration PhIP exposures were required. SA-*β*-Gal expression, western blotting, DCFDA assay, and fluorescence microscopy corroborated the adverse effects of low-level PhIP releases from the particulate PhIP. Therefore, these results may offer an early assessment platform/model to efficiently analyze the acute and chronic biological effects of aerosol exposures of PhIP and its related particles.

## Methods

### Fabrication of PhIP and PhIP@OA Aerosols

For fabrication of PhIP aerosols, nitrogen (99.9999% purity) gas (3 L min^−1^) was injected into a collison atomizer containing a 100 mL DMSO solution (276855, Sigma-Aldrich, USA) with 0.09 g PhIP (PI-30051, Pichemicals, China). DMSO in the PhIP-containing droplets from the atomizer was extracted in a diffusion dryer; thus, particulate PhIP aerosols were made. In case of PhIP@OA, 0.01 g OA (O1008, Sigma-Aldrich, USA) was further added to the PhIP-containing solution. The fabricated PhIP or PhIP@OA aerosols were then electrostatically charged (i.e., positively charged) in a corona discharge reactor with pin-ring electrodes, and the aerosols were collected on a cylindrical rod with negative potential (−3 kV, direct current voltage) in an electrostatic precipitator. The aerosol collection rod was immersed in the PBS solution under ultrasonic irradiation for 10 min to detach the aerosols from the rod. The rod was then removed from the solution and rinsed with PBS, and additional PBS was further injected into the solution to disperse PhIP or PhIP@OA with a desired mass concentration (Supplementary Fig. S1).

### Physicochemical Characterizations

A scanning mobility particle sizer (SMPS, 3936, TSI, USA) and a dynamic light scattering (DLS) particle sizer (Nano-ZS, Malvern Instruments, UK) were employed to measure particle size distributions of PhIP and PhIP@OA particles in the aerosol and aqueous states, respectively. Morphologies of the particles were analyzed using a transmission electron microscope (TEM, Tecnai G2 F20 S-TWIN, FEI, USA). To analyze chemical characteristics, PhIP and PhIP@OA aerosols were directly deposited onto polytetrafluoroethylene substrates (PTFE, 11807-47-N, Sartorius, Germany), and the particle-deposited substrates were transferred to Fourier transform infrared spectroscopy (FTIR, Nicolet iS10, Thermo Fisher Scientific, USA) for absorbance detection within the range of 3500-650 cm^−1^. A highly sensitive mercury cadmium telluride (MCT) detector was employed for microanalysis of the particles on a PTFE substrate, and the detector was cooled using liquid nitrogen during the measurements to maintain detection sensitivity.

### Biological Assays

Cytotoxicity measurements of PhIP and PhIP@OA particles, including dissolved PhIP, on SHSY5Y, MRC5, and HDF cells were conducted using MTT assays. After particle exposures, cells were incubated with 0.5 mg mL^−1^ MTT reagent for 2 h in the dark. Subsequently, the formazan was completely dissolved by adding isopropyl alcohol containing 40 mM HCl, and the absorbance of samples was measured at 570 nm using an ELISA plate reader (Thermo Multiskan Spectrum, USA). SA-*β*-gal activity in HDF cells was measured using the chromogenic cytochemical assay^32^. The cells treated with the samples including doxorubicin (DOX) and paraformaldehyde were washed with PBS and stained with 5-bromo-4-chloro-3-indolyl-*β*-D-galactopyranoside solution (B4252, Sigma-Aldrich, USA). The increased level of lysosomal *β*-gal was indicated by blue-green stains, as visualized under inverted bright-field microscopy (IX73, Olympus, USA), and SA-*β*-gal positive cells were represented as a percentage of the total cell number. For western blot analysis, the proteins on a polyvinylidene fluoride membrane from particle-exposed HDF cells were incubated with primary antibodies against glyceraldehyde-3-phosphate dehydrogenase, p21, and p53 (Santa Cruz Biotechnology, Inc., USA). The proteins were then detected using enhanced chemiluminescence agents on a luminescent image analyzer (LAS-4000 mini, Fujifilm, Japan). To measure ROS, the 2′,7′-dichlorodihydrofluorescein diacetate assay (DCFDA) (ab113851, Abcam, UK) was employed in HDF cells after treatment with the samples including DOX (Abcam, UK). The 2′,7′-dichlorofluorescein probe was visualized using fluorescence microscopy (Eclipse Ti, Nikon, Japan) and analyzed using a flow cytometer (FACSVerse, BD Biosciences, USA).

### Data availability statement

All data generated or analysed during this study are included in this published article (and its Supplementary Information file).

## Electronic supplementary material

Supplementary Information
