# Supplementary material for: In vitro exposure of simulated meat-cooking fumes to assess adverse biological effects
Source: Sci Rep. 2017 Sep 7;7:10841. doi: 10.1038/s41598-017-11502-8 (PMC5589928; doi:10.1038/s41598-017-11502-8)
Supplement: Supplementary file 1 — Supplementary Information [file 41598_2017_11502_MOESM1_ESM.pdf]

Supplementary Information for Publication

In vitro exposure of simulated meat-cooking fumes to assess adverse biological effects

Bijay Kumar Poudel[1,*], Jungwook Choi[1,*], Jae Hong Park[2], Kyung-Oh Doh[3] & Jeong Hoon Byeon[1]

[*]These authors contributed equally to this work. Corresponding authors: postjb@yu.ac.kr or park895@purdue.edu or kodoh@ynu.ac.kr

Table of Contents:

Fig. S1. Schematics of fabrications of PhIP, PhIP@OA, PhIP-CB@OA, and PhIP@DOS aerosols in a continuous single-pass configuration.

Fig. S2. Particle size distributions of CB and PhIP-CB@OA aerosols.

Fig. S3. Particle size distributions (measured using SMPS) of PhIP, DOS, and PhIP@DOS aerosols.

Fig. S4. TEM images of CB, PhIP-CB@OA NPs, and PhIP@DOS NPs (High-resolution TEM showing the characteristic lattice fringes of CB, OA, DOS and PhIP parts on the right).

Fig. S5. FTIR spectra for fabricated aerosols.

Fig. S6. Particle size distributions of PhIP, PhIP@OA, PhIP-CB@OA, and PhIP@DOS particles in PBS solution.

Fig. S7. MTT assay results for (a) PhIP-CB@OA aerosols on SHSY5Y, MRC5, and HDF cells after a 48 h exposure, and for (b) PhIP, PhIP@OA, and PhIP@DOS aerosols on HDF cells after a 96 h exposure.

Fig. S8. Schematic for different responses of tested cells due to different PhIP phases (dissolved PhIP vs particulate PhIP). The HPLC (LC-4000, Jasco Inc., USA) results are included as an inset, and fluorescence was monitored at 316 nm (excitation) and 370 nm (emission) with a Dynamax FL-1 detector (Rainin, USA).

Fig. S9. Western blot analysis of DOX-, PhIP-, and PhIP@OA-treated HDF cells.

Table S1. Mass fractions [measured using a piezobalance particle monitor (3522, Kanomax, Japan)] of PhIP, OA, CB, and DOS in each sample.

Table S2. Summaries of SMPS measurements of PhIP, OA, and PhIP@OA aerosols.

Table S3. Summaries of SMPS measurements of CB, PhIP-CB@OA, DOS, and PhIP@DOS aerosols.

Table S4. Zeta potentials of PhIP, PhIP@OA, PhIP-CB@OA, and PhIP@DOS particles dispersed in PBS solution (pH 7.4), including "Dissolved PhIP" as PhIP molecules in solution.

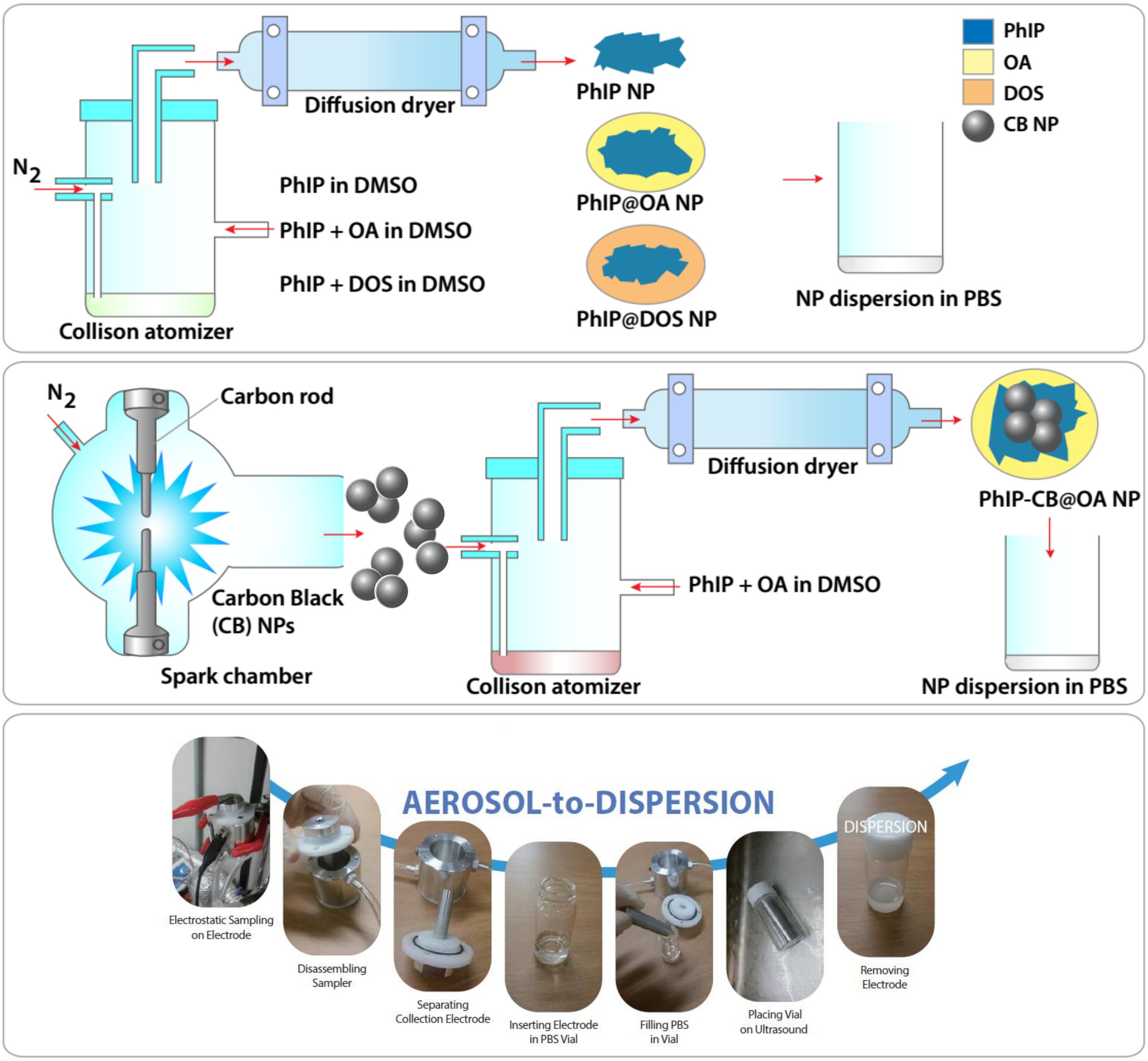

Fig. S1. Schematics of fabrications of PhIP, PhIP@OA, PhIP-CB@OA, and PhIP@DOS aerosols in a continuous single-pass configuration. To prepare PhIP@DOS aerosols, 0.01 g DOS was added to the PhIP-containing solution instead of OA. In case of PhIP-CB@OA, CB particles were first prepared by spark ablation, where a direct high voltage current (3 kV) was applied between two graphite rods (C-072561, Nilaco, Japan) to induce spark ablation in the presence of nitrogen gas flow (3 L min^{-1}). The vaporized graphite from spark ablation was subsequently condensed into CB particles by the gas flow. The CB particle-laden flow was directly injected into the collison atomizer filled with the PhIP@OA solution. Hybrid droplets from the atomizer were then passed through a diffusion dryer to extract the DMSO solvent from the droplets, resulting in the formation of PhIP-CB@OA aerosols. The fabricated aerosols were dispersed in PBS (the particle recovery efficiency from the collection electrode was approximately 91.6%, mass basis) and then compared with dissolved PhIP by in vitro assays (MTT, SA-β-gal, western blot, and flow cytometry).

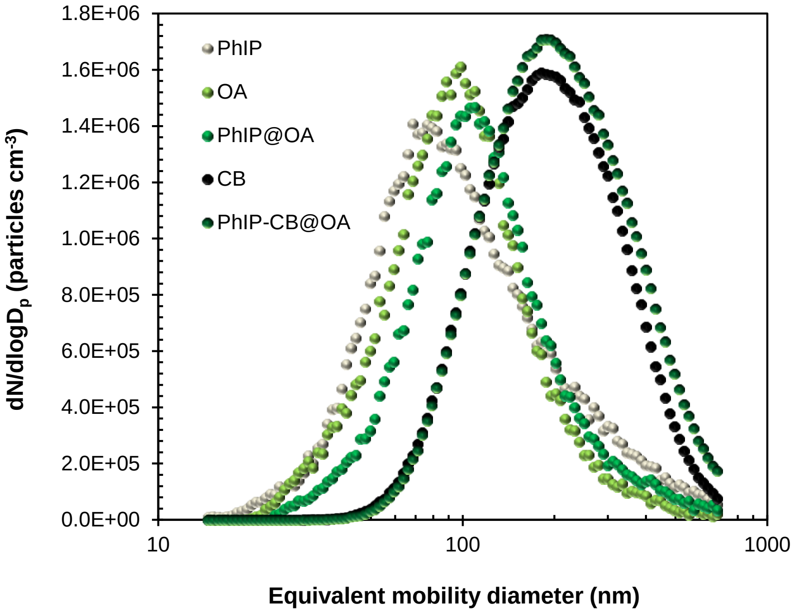

Fig. S2. Particle size distributions of CB and PhIP-CB@OA aerosols. Size distributions were measured using SMPS, which comprised a differential mobility analyzer, condensation particle counter, and aerosol charge neutralizer. The size distributions of PhIP, OA, and PhIP aerosol are co-displayed for comparative purposes.

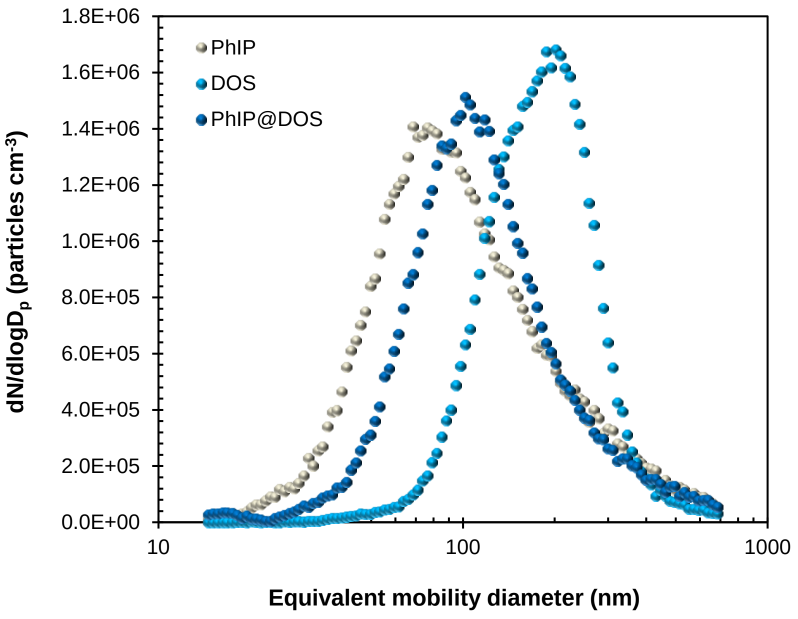

Fig. S3. Particle size distributions (measured using SMPS) of PhIP, DOS, and PhIP@DOS aerosols.

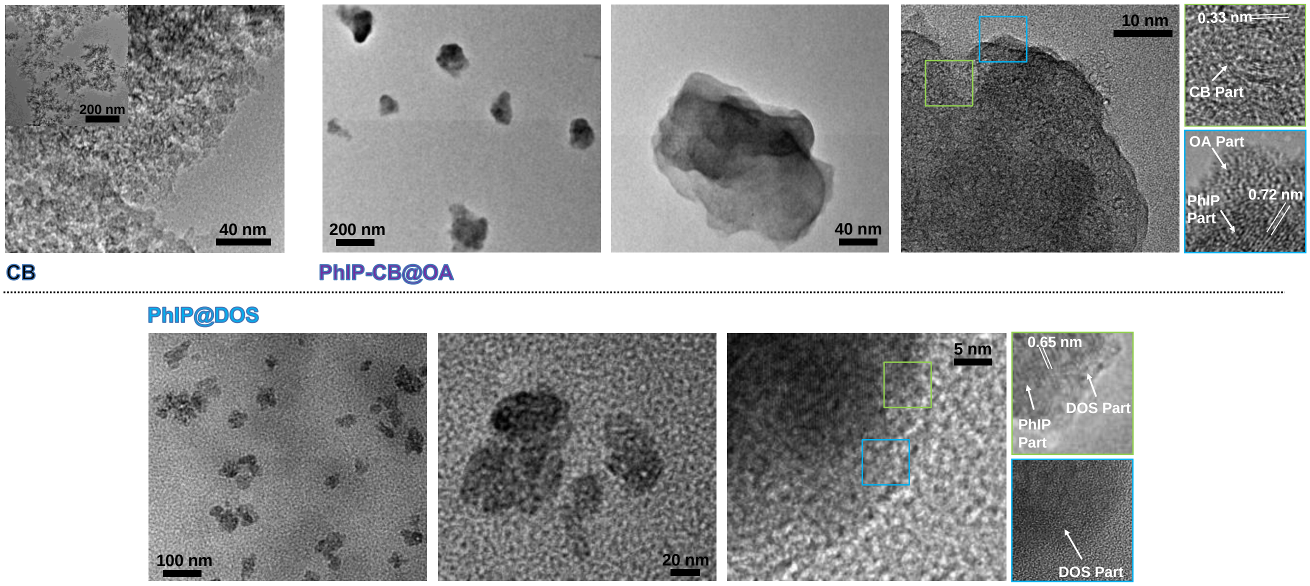

Fig. S4. TEM images of CB, PhIP-CB@OA NPs, and PhIP@DOS NPs (High-resolution TEM showing the characteristic lattice fringes of CB, OA, DOS and PhIP parts on the right).

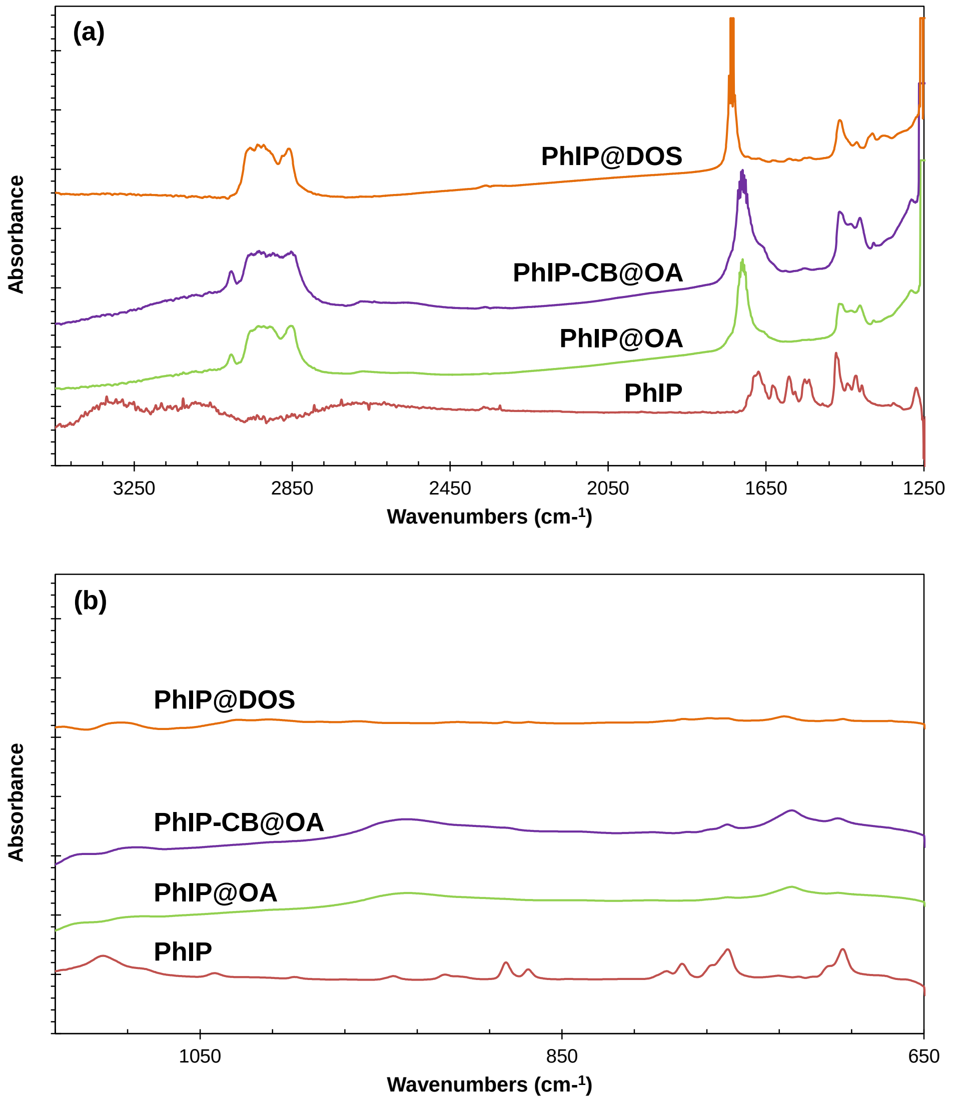

Fig. S5. FTIR spectra for fabricated aerosols. PhIP, PhIP@OA, PhIP-CB@OA, and PhIP@DOS particles were deposited directly on polytetrafluoroethylene (PTFE) substrates via mechanical filtration. The spectra are divided as (a) 1250–3500 cm^{-1} and (b) 650–1100 cm^{-1}.

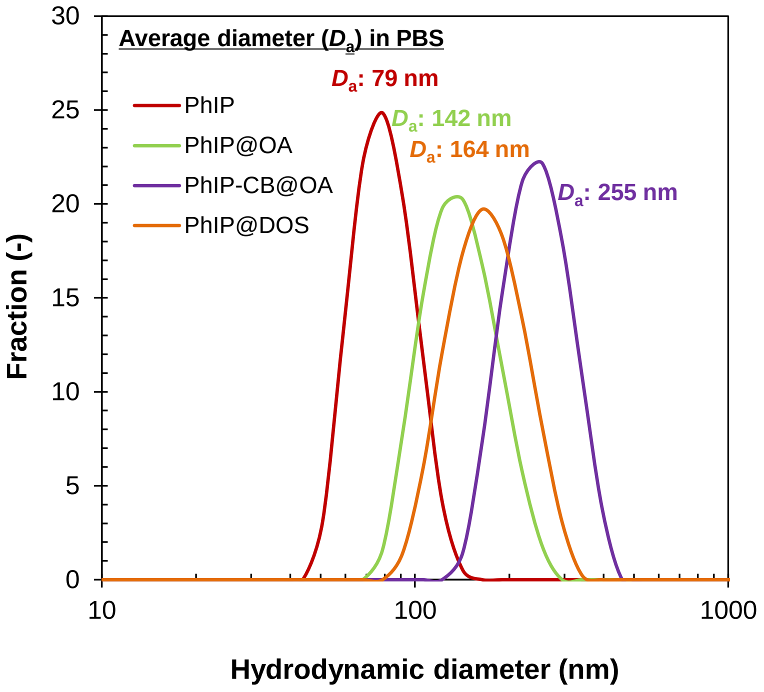

Fig. S6. Particle size distributions of PhIP, PhIP@OA, PhIP-CB@OA, and PhIP@DOS particles in PBS solution. The fabricated aerosols were first collected on an electrode in an electrostatic precipitator. The electrode then was immersed in PBS, releasing the collected aerosol to form dispersions. Size distributions were measured using a DLS particle size analyzer.

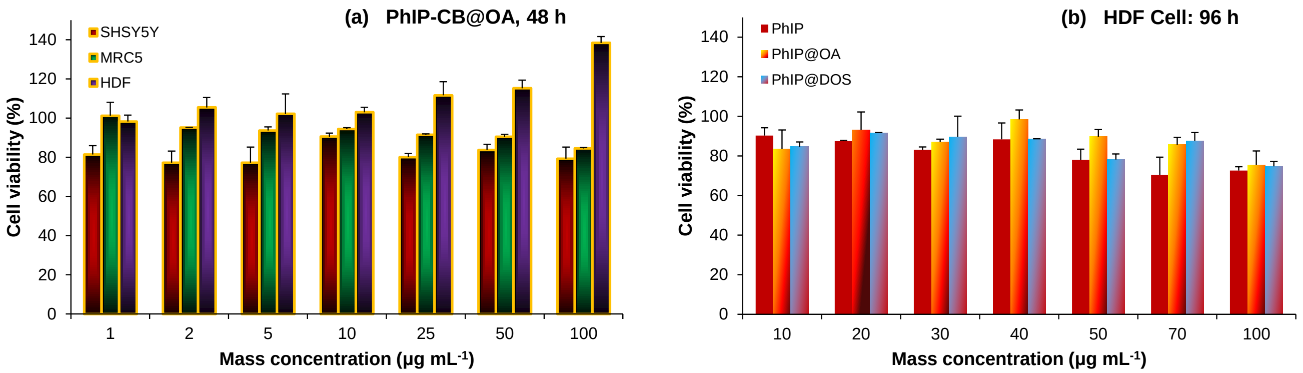

Fig. S7. MTT assay results for (a) PhIP-CB@OA aerosols on SHSY5Y, MRC5, and HDF cells after a 48 h exposure, and for (b) PhIP, PhIP@OA, and PhIP@DOS aerosols on HDF cells after a 96 h exposure.

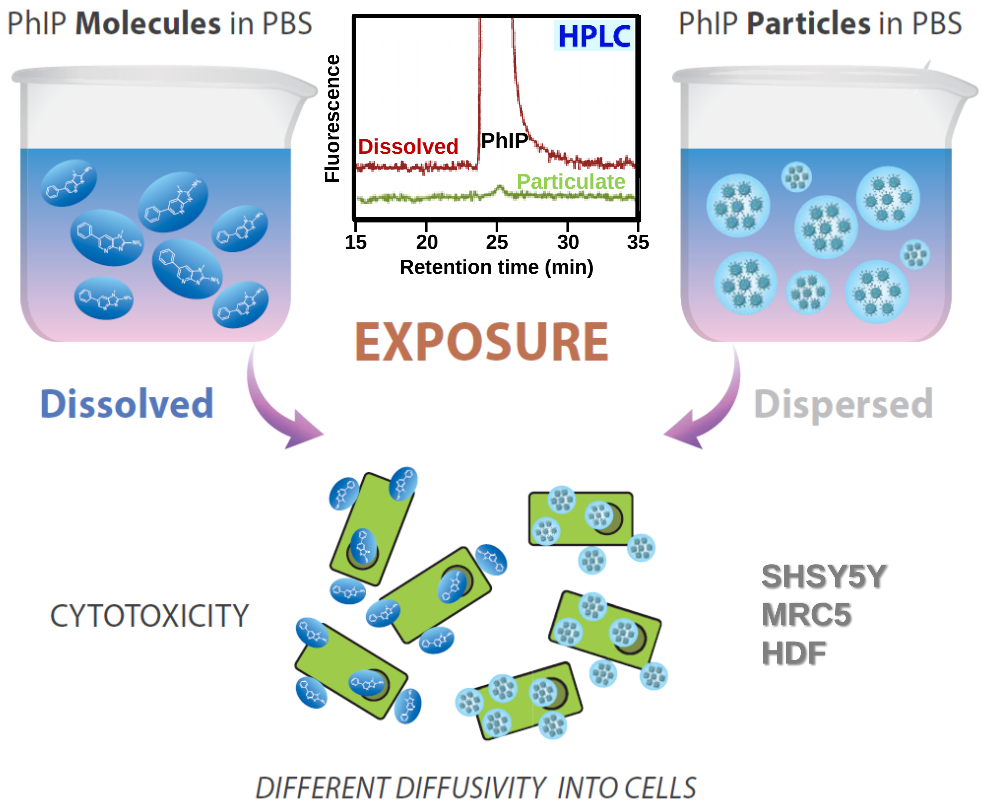

Fig. S8. Schematic for different responses of tested cells due to different PhIP phases (dissolved PhIP vs particulate PhIP). The HPLC (LC-4000, Jasco Inc., USA) results are included as an inset, and fluorescence was monitored at 316 nm (excitation) and 370 nm (emission) with a Dynamax FL-1 detector (Rainin, USA). The results support the significantly reduced elution (retention time at ~24 min) of PhIP molecules from the particulate PhIP.

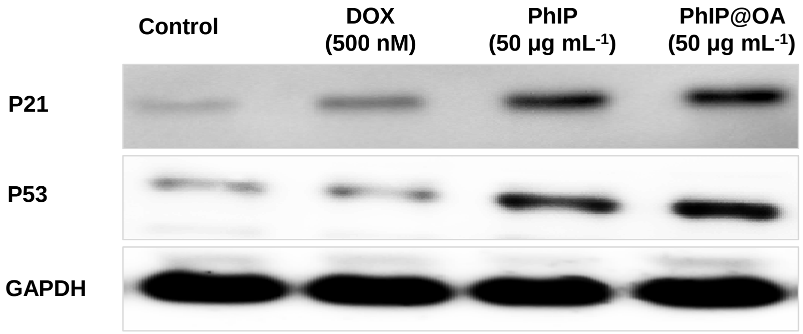

Fig. S9. Western blot analysis of DOX-, PhIP-, and PhIP@OA-treated HDF cells.

Table S1 Mass fractions [measured using a piezobalance particle monitor (3522, Kanomax, Japan)] of PhIP, OA, CB, and DOS in each sample

Case	PhIP	OA	CB	DOS
	Mass fraction (-)			
PhIP	1.000	-	-	-
PhIP@OA	0.900	0.100	-	-
PhIP-CB@OA	0.750	0.085	0.165	-
PhIP@DOS	0.782	-	-	0.218

Table S2 Summaries of SMPS measurements of PhIP, OA, and PhIP@OA aerosols

Case	Geometric mean diameter	Geometric standard deviation	Total number concentration
	nm	-	particles cm^{-3}
PhIP	66.5	1.89	1.2×10^6
OA	87.3	1.93	8.7×10^5
PhIP@OA	98.5	1.73	8.9×10^5

Table S3 Summaries of SMPS measurements of CB, PhIP-CB@OA, DOS, and PhIP@DOS aerosols

Case	Geometric mean diameter	Geometric standard deviation	Total number concentration
	nm	-	particles cm^{-3}
CB	191.1	1.66	9.5×10^5
PhIP-CB@OA	201.9	1.69	1.0×10^6
DOS	176.2	1.51	7.3×10^5
PhIP@DOS	115.7	1.78	7.8×10^5

Table S4 Zeta potentials of PhIP, PhIP@OA, PhIP-CB@OA, and PhIP@DOS particles dispersed in PBS solution (pH 7.4), including "Dissolved PhIP" as PhIP molecules in solution

Case	Zeta potential
	mV
Dissolved	-16.2±1.2
PhIP	-17.7±1.0
PhIP@OA	-16.8±1.4
PhIP-CB@OA	-12.2±1.9
PhIP@DOS	-17.4±0.8

at pH 7.4
